# Comparison of consumer knowledge about *Campylobacter, Salmonella* and *Toxoplasma* and their transmissibility via meat: results of a consumer study in Germany

**DOI:** 10.1186/s12889-020-08476-0

**Published:** 2020-03-16

**Authors:** K. A. Henke, T. Alter, M. G. Doherr, R. Merle

**Affiliations:** 1 Institute for Veterinary Epidemiology and Biostatistics, Freie Universität Berlin, Königsweg 67, 14163 Berlin, Germany; 2 Institute of Food Safety and Food Hygiene, Freie Universität Berlin, Königsweg 67 and 69, 14163 Berlin, Germany

**Keywords:** Campylobacter, Salmonella, Toxoplasma, Public health, Online survey, Awareness

## Abstract

**Background:**

*Campylobacter* is the most commonly reported causative agent of foodborne bacterial infection in Germany, and contaminated chicken meat is an important source of this zoonotic agent. The aim of this study was to determine the knowledge of consumers in Germany about *Campylobacter, Salmonella and Toxoplasma* and their transmissibility via meat. In addition, we investigated the level of knowledge between selected consumer groups and whether the results coincided with those of international studies.

**Methods:**

We conducted a cross-sectional survey of 1008 consumers in Germany via an online panel to record, analyse and evaluate the state of knowledge about *Campylobacter, Salmonella* and *Toxoplasma*. The participants were selected according to age, gender and federal states to be representative of the German population.

**Results:**

Overall, 68.3% of the respondents had never heard of *Campylobacter*, 20.2% had heard of *Campylobacter* but did not know how to protect themselves, and only 11.5% knew how to protect themselves from *Campylobacter* infections. Slightly more than half (52.2%) of the respondents who had at least heard of *Campylobacter* knew that *Campylobacter* was transmissible via meat. Knowledge increased significantly with age. Participants over 60 years old knew about *Campylobacter* almost three times as often as the 16- to 19-year-old comparison group (OR = 2.982). Consumers who had at least a secondary school certificate were almost twice as likely to know about *Campylobacter* as those who had no school certificate or a lower secondary school certificate (OR = 1.899). Participants who were not actors in the food chain were significantly less frequently informed about *Campylobacter* than were those who were actors in the food chain. Consumer knowledge of *Toxoplasma* was better than that of *Campylobacter*. Consumers have the most knowledge about *Salmonella*.

**Conclusions:**

Consumers in Germany are predominantly poorly informed about *Campylobacter* and the transmission route via meat. General knowledge of *Toxoplasma* is better than that of *Campylobacter*. Among the three pathogens, consumers are best informed about *Salmonella*. This finding highlights the importance of making existing information materials more accessible to consumers in the future to increase their knowledge, with the objective of reducing the incidence of *Campylobacter* infections.

## Background

*Campylobacter, Salmonella* and *Toxoplasma* are zoonotic agents that can be transmitted via food [1]. Foodborne infections occur in most countries worldwide, although with various levels of reported cases. Countries differ, for example, in that they either have compulsory and voluntary reporting systems. In addition, either case-related or aggregated data are made publicly available [2]. Germany is one of the countries stringent rules on food hygiene and a well-implemented public health system with regular testing of stool samples in suspected cases. With 69,414 confirmed cases in 2017 [3], enteritis caused by *Campylobacter* is the most common bacterial infection causing diarrhoeal disease reportable in Germany. In comparison, the 14,269 confirmed cases of salmonellosis are low. Nevertheless, it is, after campylobacter enteritis, the second-most common reportable bacterial gastrointestinal disease [3]. Seven cases of congenital human toxoplasmosis were confirmed in 2017. The recorded incidence of *Campylobacter* was 84 cases per 100,000 inhabitants in 2017. For salmonellosis, there were 17 cases per 100,000 inhabitants [3]. The average notification rate within the European Union was 64.8 cases of *Campylobacter* per 100,000 inhabitants in 2017. The highest country-specific notification rates in 2017 were observed in the Czech Republic (230.0 cases per 100,000), Slovakia (127.8), Sweden (106.1) and Luxembourg (103.8). In 2017, the lowest rates were observed in Bulgaria, Cyprus, Latvia, Poland, Portugal and Romania (≤5.8 per 100,000) [2]. In New Zealand, the incidence of *Campylobacter* of 158.9 cases per 100,000 inhabitants in 2016 was almost twice as high as that in Germany [4]*.* Compared to *Campylobacter*, the reporting rate within the European Union indicates that there were 19.7 cases of salmonellosis per 100,000 inhabitants in the same year and 1.31 cases of toxoplasmosis per 100,000 live births [2]. An infection with *Salmonella* or *Campylobacter* can result in acute common unspecific symptoms such as diarrhoea, abdominal pain, fever and vomiting [3]. In addition to acute unspecific symptoms, complications such as Guillain-Barré syndrome and reactive arthritis can occur as long-term sequelae of *Campylobacter* [5]. Infection with *Toxoplasma* is usually subclinical in healthy adults, but an initial infection during pregnancy can result in severe damage, e.g., to the brain of the unborn child [3]. A common source of *Campylobacter* infection is poultry meat, especially broiler meat [2]. Beef and pork can also be considered sources, but red meat is far less likely to be contaminated with *Campylobacter* than poultry meat [6]. A common source of *Salmonella* infections are eggs and egg products, meat and meat product subcategories and bakery products [2]. The transmission of *Toxoplasma* may occur through insufficiently cooked contaminated meat or handling of infected cats [3].

According to different studies, the majority of consumers in Germany believe that food is 100% safe [7, 8]. Although 23% of consumers in Germany are aware that *Campylobacter* may be present in food, only 9% are concerned about the possibility of acquiring foodborne campylobacteriosis [9]. In general, consumer awareness of specific pathogens has increased [10], but international studies have also indicated that consumers’ knowledge about *Campylobacter* is predominantly poor [11–16]. Although consumers generally know that microorganisms such as *Salmonella* can be present in meat and may cause food-borne diseases [17, 18], it becomes apparent that many consumers are unaware that *Campylobacter* is explicitly transmitted via meat [16–19].

According to the European Food Safety Authority, between 20 and 30% of *Campylobacter* cases in humans can be attributed to the handling, preparation and consumption of chicken meat [20]. However, there is insufficient evidence to show how much knowledge consumers or certain consumer groups in Germany have about *Campylobacter* and whether they are aware that *Campylobacter* can be transmitted directly or indirectly via meat.

The aim of the study was therefore to assess the knowledge of consumers in Germany about *Campylobacter* and its transmission pathways and to compare this knowledge to that about *Salmonella* and *Toxoplasma*. Differences in knowledge between selected consumer groups were identified to better target information campaigns. In addition, the general knowledge of consumers on *Campylobacter*, *Salmonella* and *Toxoplasma* were compared. In our view, more knowledge about the pathways of infection at the consumer level is essential to improve public health.

## Methods

### Data collection and questionnaire development

To conduct this study, a questionnaire was designed comprising a total of 43 questions divided into five sections. The questionnaire focused not only on *Campylobacter* but also included other zoonotic pathogens transmissible via meat, such as *Salmonella* and *Toxoplasma*. For comparison purposes, pathogens such as the rabies virus and the human immunodeficiency virus, which are not transmissible to humans via meat, were also included. Questions covered the following topics: sociodemographic and socioeconomic factors; consumer knowledge of pathogens such as *Campylobacter, Salmonella* and *Toxoplasma*; involvement; and the influence of selected actors in the poultry meat food chain on poultry meat safety and quality from the consumer perspective. The participants of the study were registered consumers in an online access panel in Germany who were invited to participate in the online survey via an individual e-mail link. This link could only be used once for participation; thus, multiple participation sessions were excluded. The questionnaire was written in German, and an English translation is available in the supplements. The panel provider *GapFish* recommended surveying at least 1000 consumers in Germany to obtain a good demographic representation and ensure a low margin of error. *GapFish* is a company and the operator of a panel platform where participants are registered and can be selected according to personal data such as age, gender and occupation. The target group was the population aged 16 years and older. Only persons with German language skills could participate, which might have partly excluded first-generation immigrants. To ensure representativeness regarding age and geographical location, the study population was proportionally stratified according to federal state, gender and age group. Sampling was continued until all strata were complete. If a stratum was complete, further participation was refused. Ultimately, responses from 1008 consumers were included in the analysis. Data collection started on 11 August 2017 and was completed on 20 August 2017.

### Statistical analysis

The evaluation of the data was conducted with IBM SPSS Statistics Version 24. The responses of 1008 consumers who had answered all questions completely were included in the statistical analysis. We did not have to remove any incomplete answers, as 1008 complete answers were submitted directly by the panel provider. Exceptions from completeness were made regarding questions on the level of education, primary residence, number of children in the household and household income after tax. There was an option for participants to indicate that they could not answer the question or did not want to answer it. If a participant selected one of these answer options, this answer was not considered in the univariable and multivariable analyses, thus reducing the item-specific sample size.

Descriptive statistics included frequency tables concerning questions about *Salmonella*, *Campylobacter* and *Toxoplasma*.

Univariable associations between categorical variables were analysed by cross-tabulation and chi-square statistics. If the number of cells with expected values below 5 was above 25%, Fisher’s exact test was used instead of the chi-square test. The level of significance was set to 0.05. Whenever possible, odds ratios (ORs) were calculated to compare the odds of a certain event in one group to those in other group. First, three different target variables were examined. In the first set of chi-square tests, the target variable was defined as the general level of knowledge of *Campylobacter* among consumers. The parameter values of these target variables were “*Campylobacter* is unknown” vs. “*Campylobacter* is known”. The influence of various factors, such as age and gender, on the probability that the participant had heard of *Campylobacter* was investigated. In the second model, consumer knowledge of the transferability of *Campylobacter* via meat was used as the dependent variable. The parameter values of these target variables were “transferability is known” vs. “transferability is not known”. These questions could only be answered by consumers who had already heard of *Campylobacter*. Here, the influence of various factors, such as age and gender, on the likelihood that the participant knew that *Campylobacter* could be transmitted to humans via meat was investigated. Third, it was investigated whether the different levels of consumer knowledge about possible protective measures against *Campylobacter* had an influence on the likelihood that consumers were aware of the transferability of *Campylobacter* via meat. The parameter values of knowledge were “*Campylobacter* is known, but it is unknown how to protect oneself” vs. “*Campylobacter* is known and how to protect oneself”. Consumers who had never heard of *Campylobacter* before were not asked this question and therefore could not be included in the analysis. The latter test was also performed for *Salmonella* and *Toxoplasma*.

Categorical variables were analysed separately in the univariable chi-square tests and were then included in subsequent multivariable logistic regression models. This procedure was only conducted for the first two target variable analyses. The final models were identified through a manual backward selection process. In each step, the variable with the highest *p*-value was removed. After the removal of one variable, the change in the regression coefficients of the remaining variables as well as the change of model R-squared were investigated. If the changes were above 15%, the removed variable was included again to control for confounding. The final models included the variables with *p*-values < 0.05. Two-way interactions between explanatory variables were considered in the multivariable model and were removed, since all interactions turned out to be not statistically significant. Regression coefficients, *p*-values and ORs (including 95% confidence intervals) are reported.

## Results

### General knowledge about campylobacter, Salmonella and toxoplasma

Overall, 68.3% (688/1008) of the respondents had never heard of *Campylobacter*. A total of 20.2% (204/1008) had heard of *Campylobacter* but did not know how to protect themselves, while 11.5% said they knew how to protect themselves from *Campylobacter*. A total of 2.8% (28/1008) of respondents had never heard of *Salmonella*. A total of 19.9% (201/1008) had heard of *Salmonella* but did not know how to protect themselves, while 77.3% (779/1008) said they had heard of *Salmonella* and knew how to protect themselves. Of all respondents, almost half (48.3%) did not know about *Toxoplasma*. Almost ^1^/_3_ (32.8%) knew about *Toxoplasma* but did not know how to protect themselves, while 18.8% stated that they knew about both the infectious agent and how to protect themselves.

### General knowledge about *Campylobacter* from different consumer groups

An overview of general knowledge about *Campylobacter*, *Salmonella* and *Toxoplasma* from different consumer groups is shown in Table 1.

**Table 1:** Comparison of knowledge between consumer groups about *Campylobacter*, *Salmonella* and *Toxoplasma*

	*n* = 100% total	n (%) *Salmonella* is			n (%) *Campylobacter* is			n (%) *Toxoplasma* is		
		Unknown	Known, but I don’ t know how to protect myself	Known and I know how to protect myself	Unknown	Known, but I don’ t know how to protect myself	Known and I know how to protect myself	Unknown	Known, but I don’ t know how to protect myself	Known and I know how to protect myself
**Gender**										
Female	500	11 (2.2)	93 (18.6)	396 (79.2)	336 (67.2)	103 (20.6)	61 (12.2)	215 (43.0)	168 (33.6)	117 (23.4)
Male	508	17 (3.3)	108 (21.3)	383 (75.4)	352 (69.3)	101 (19.9)	55 (10.8)	272 (53.5)	163 (32.1)	73 (14.4)
**Age group**										
16–19 years old	176	16 (9.1)	63 (35.8)	97 (55.1)	141 (80.1)	18 (10.2)	17 (9.7)	135 (76.7)	21 (11.9)	20 (11.4)
20–39 years old	219	8 (3.7)	46 (21.0)	165 (75.3)	151 (68.9)	44 (20.1)	24 (11.0)	98 (44.7)	70 (32.0)	51 (23.3)
40–59 years old	451	2 (0.4)	70 (15.5)	379 (84.0)	293 (65.0)	103 (22.8)	55 (12.2)	182 (40.4)	178 (39.5)	91 (20.2)
> 60 years old	162	2 (1.2)	22 (13.6)	138 (85.2)	103 (63.6)	39 (24.1)	20 (12.3)	72 (44.4)	62 (38.3)	28 (17.3)
**Federal state**										
Eastern Germany	215	2 (0.9)	34 (15.8)	179 (83.3)	143 (66.5)	50 (23.3)	22 (10.2)	83 (38.6)	83 (38.6)	49 (22.8)
Western Germany	793	26 (3.3)	167 (21.1)	600 (75.7)	545 (68.7)	154 (19.4)	94 (11.9)	404 (50.9)	248 (31.3)	141 (17.8)
**Cold cut consumption/week**										
No consumption (never)	47	4 (8.5)	7 (14.9)	36 (76.6)	37 (78.7)	6 (12.8)	4 (8.5)	27 (57.4)	9 (19.1)	11 (23.4)
Rare to frequent (< 1/week up to 3–4/week)	582	20 (3.4)	119 (20.4)	443 (76.1)	403 (69.2)	113 (19.4)	66 (11.3)	285 (49.0)	185 (31.8)	112 (19.2)
Very common (5–6/week or daily)	379	4 (1.1)	75 (19.8)	300 (79.2)	248 (65.4)	85 (22.4)	46 (12.1)	175 (46.2)	37 (36.1)	67 (17.7)
**Meat consumption/week**										
No consumption (never)	35	2 (5.7)	4 (11.4)	29 (82.9)	25 (71.4)	7 (20.0)	3 (8.6)	17 (48.6)	10 (28.6	8 (22.9)
Rare to frequent (< 1/week up to 3–4/week)	784	19 (2.4)	148 (18.9)	617 (78.7)	546 (69.6)	151 (19.3)	87 (11.1)	390 (49.7)	255 (32.5)	139 (17.7)
Very common (5-6x/week or daily)	379	7 (3.7)	49 (25.9)	133 (70.4)	117 (61.9)	46 (24.39)	26 (13.8)	80 (42.3)	66 (34.9)	43 (22.8)
**Level of education**										
No certificate or lower secondary school certificate	108	4 (3.7)	28 (25.9)	76 (70.4)	85 (78.7)	13 (12.0)	10 (9.3)	71 (65.7)	28 (25.9)	9 (8.3)
Secondary school certificate	878	23 (2.6)	165 (18.8)	690 (78.6)	587 (66.9)	188 (21.4)	103 (11.7)	404 (46.0)	298 (33.9)	176 (20.0)
**Size of the main residence (number of inhabitants)**										
Rural community (< 5000 inhabitants)	158	2 (1.3)	36 (22.8)	120 (75.9)	111 (70.3)	28 (17.7)	19 (12.0)	68 (43.0)	53 (33.5)	37 (23.4)
Small-size town (between 5000 and less than 20,000)	220	10 (4.5)	47 (21.4)	163 (74.1)	157 (71.4)	40 (18.2)	23 (10.5)	113 (51.4)	70 (31.8)	37 (16.8)
`Mid-size town (between 20,000 and less than 100,000)	264	7 (2.7)	54 (20.5)	203 (76.9)	175 (66.3)	58 (22.0)	31 (11.7)	124 (47.0)	94 (35.6)	46 (17.4)
Metropolis (100,000 and more)	332	6 (1.8)	55 (16.6)	271 (81.6)	220 (66.3)	75 (22.6)	37 (11.1)	161 (48.5)	108 (32.5)	63 (19.0)
**Children in the household**										
At least 1 child	337	17 (5.0)	87 (25.8)	233 (69.1)	229 (68.0)	67 (19.9)	41 (12.2)	162 (48.1)	89 (26.4)	86 (25.5)
No children	654	11 (1.7)	109 (16.7)	534 (81.7)	444 (67.9)	136 (20.8)	74 (11.3)	315 (48.2)	236 (36.1)	103 (15.7)
**Monthly household income after taxes**										
Low income (< 1500€)	252	12 (4.8)	62 (24.6)	178 (70.6)	179 (71.0)	46 (18.3)	27 (10.7)	133 (52.8)	81 (32.1)	38 (15.1)
Middle income (1500 < 3600€)	418	7 (1.7)	65 (15.6)	346 (82.8)	277 (66.3)	93 (22.2)	48 (11.5)	177 (42.3)	155 (37.1)	86 (20.6)
High income (> 3600€)	176	1 (0.6)	33 (18.8)	142 (80.7)	111 (63.1)	44 (25.0)	21 (11.9)	75 (42.6)	62 (35.2)	39 (22.2)
**Occupational groups**										
Actor in the food chain	117	7 (6.0)	25 (21.4)	85 (72.6)	64 (54.7)	31 (26.5)	22 (18.8)	54 (46.2)	34 (29.1)	29 (24.8)
Not an actor in the food chain	891	21 (2.4)	176 (19.8)	694 (77.9)	624 (70.0)	173 (19.4)	94 (10.5)	433 (48.6)	297 (33.3)	161 (18.1)
**Veterinarian**										
Yes	12	1 (8.3)	4 (33.3)	7 (58.3)	2 (16.7)	4 (33.3)	6 (50.0)	3 (25.0)	4 (33.3)	5 (41.7)
No	996	27 (2.7)	197 (19.8)	772 (77.5)	686 (68.9)	200 (20.1)	110 (11.0)	484 (48.6)	327 (32.8)	185 (18.6)

A total of 67.2% (336/500) of the women and 69.3% (352/508) of the men did not know about *Campylobacter*. A total of 20.6% (103/500) of women did not know how to protect themselves from infection; these women differed from those who had not heard of it. A total of 19.9% of men (101/508) did not know how to protect themselves from infection; these men also differed from those who had not heard of it. Approximately 12.2% (61/500) of women and 10.8% (55/508) of men knew how to protect themselves.

Of the young adults between 16 and 19 years of age, only 10.2% (18/176) knew about *Campylobacter* but did not know how to protect themselves, and 9.7% (17/176) both knew about *Campylobacter* and knew how to protect themselves. Of the 20- to 39-year-old participants, 20.1% (44/219) knew about *Campylobacter* but did not know how to protect themselves, while 11.0% (24/219) said they knew how to protect themselves from infection. Of those consumers with no or a lower secondary school certificate, 12% (13/108) had already heard of *Campylobacter,* and 9.3% (10/108) also knew how to protect themselves from infection. Of those who had at least a secondary school certificate, 21.4% (188/878) knew about *Campylobacter* but did not know how to protect themselves, and 11.7% (103/878) knew how to protect themselves.

Of all respondents, 11.6% (117/1008) said they worked actively in the food chain. This included agricultural holdings, meat sales, slaughtering and processing, food monitoring and animal transport. Approximately half (64/117) of these respondents did not know about *Campylobacter,* 26.5% (31/117) said they had heard of *Campylobacter*, and 18.8% (22/117) indicated that they knew how to protect themselves from *Campylobacter*. Of the participating veterinarians, 16.7% (2/12) did not know about *Campylobacter*, 33.3% (4/12) knew about *Campylobacter* but did not know how to protect themselves against infection, and 50.0% (6/12) knew how to protect themselves.

### General knowledge of *Salmonella* from different consumer groups

Of the young adults between 16 and 19 years of age, 35.8% (63/176) knew about *Salmonella* but did not know how to protect themselves, and 55.1% (97/176) knew about *Salmonella* and how to protect themselves. Of the > 60-year-old participants, 13.6% (22/162) knew about *Salmonella* but did not know how to protect themselves, while 85.2% (138/162) said they knew how to protect themselves from infection.

Approximately ¼ (24.6%) of low-income respondents (62/252) knew about *Salmonella* but did not know how to protect themselves, and 70.6% (178/252) of this group knew how to protect themselves from infection. Among consumers with high incomes, 18.8% (33/176) did not know how to protect themselves, while 80.7% from this group knew how to protect themselves. Of those consumers who were actors in the food chain, 6% did not know about *Salmonella*, while 72.6% knew how to protect themselves. Of consumers who were not actors in the food chain, 2.4% were unaware of *Salmonella*. On the other hand, 77.9% of them knew how to protect themselves.

### General knowledge of *Toxoplasma* from different consumer groups

Of the young adults between 16 and 19 years, 76.7% (135/176) did not know about *Toxoplasma.* Of the adults between 20 and 39 years of age, 44.7% (98/219) did not know about Toxoplasma. In all age groups, less than 25% know how to protect themselves from toxoplasmosis infection. Of the women, 43.0% did not know about *Toxoplasma*, and 53.5% did know about Toxoplasma. Almost 25% of female responders knew how to protect themselves from infection. The percentage for men was 14.4%. Of the respondents with children (162/337) and those without (315/654), almost the same number (48.1 and 48.2%) did not know about toxoplasma. Approximately one-quarter of respondents with children (25.5%) knew how to protect themselves from infection. Of those without children, 15.7% knew this information.

### Consumer knowledge about meat as a vector of *Campylobacter*, *Salmonella* and *Toxoplasma*

Consumers who had at least indicated knowing about *Campylobacter, Salmonella* or *Toxoplasma* were asked if the respective pathogen was transmissible via meat. Slightly more than half (52.2%) of respondents who had at least heard of *Campylobacter* (167/320) said that *Campylobacter* was transmissible via meat.

Of those consumers who did not know how to protect themselves against *Campylobacter* infection, 45.6% (93/204) thought *Campylobacter* could be transmitted via meat. In comparison, 63.8% (74/116) of consumers who knew how to protect themselves against *Campylobacter* infection thought that *Campylobacter* could be transmitted via meat (Table 2). This difference was statistically significant (OR = 2.1; *p* = 0.002, chi-square test).

**Table 2:** General consumer knowledge about meat as a vector of Campylobacter based on a representative survey in Germany (2017)

Consumer knowledge	n	n (%) *Campylobacter* is transmissible via meat	n (%) *Campylobacter* is not transmissible via meat	X^2^ test *p*-value	OR	95% CI
I have heard of it, **but I do not know** how to protect myself.	204	93 (45.6)	111 (54.4)	**0.002**	2.1	1.32–3.36
I have heard about it, and **I know** how to protect myself.	116	74 (63.8)	42 (36.2)			
Total	320	167 (52.2)	153 (47.8)			

Of those consumers who did not know how to protect themselves against *Salmonella* infection, 78.6 (158/201) thought that *Salmonella* could be transmitted via meat. In comparison, 88.7% (691/779) of consumers who knew how to protect themselves against *Salmonella* infection thought that *Salmonella* could be transmitted via meat (Table 3). This difference was statistically significant (OR = 2.1; *p* <  0.001, chi-square test).

**Table 3:** General consumer knowledge about meat as a vector of *Salmonella* based on a representative survey in Germany (2017)

Consumer knowledge	n	n (%) *Salmonella i*s transmissible via meat	n (%) *Salmonella* is not transmissible via meat	X^2^ test *p*-value	OR	95% Cl
I have heard of it, **but I do not know** how to protect myself.	201	158 (78.6)	43 (21.4)	**< 0.001**	2.1	1.43–3.20
I have heard about it, and **I know** how to protect myself.	779	691 (88.7)	88 (11.3)			
Total	980	849 (86.6)	131 (13.4)			

Approximately half (50.7%) of respondents who had at least heard of *Toxoplasma* (257/521) said that *Toxoplasma* was transmissible via meat. Of those consumers who did not know how to protect themselves against *Toxoplasma* infection, 40.8 (135/331) thought that *Toxoplasma* could be transmitted via meat. In comparison, 64.2% (122/190) of consumers who knew how to protect themselves against *Toxoplasma* infection thought that *Toxoplasma* could be transmitted via meat (Table 4). This difference was statistically significant (OR = 2.6; *p* <  0.001, chi-square test).

**Table 4:** General consumer knowledge about meat as a vector of *Toxoplasma* based on a representative survey in Germany (2017)

Consumer knowledge	n	n (%) *Toxoplasma* is transmissible via meat	n (%) *Toxoplasma* is not transmissible via meat	X^2^ test *p*-value	OR	95% CI
I have heard of it, **but I do not know** how to protect myself.	331	135 (40.8)	196 (59.2)	**< 0.001**	2.6	1.80–3.77
I have heard about it, and **I know** how to protect myself.	190	122 (64.2)	68 (35.8)			
Total	521	257 (50.7)	264 (49.3)			

### Investigation of knowledge about *Campylobacter* within different consumer groups

The level of knowledge varied significantly between different consumer groups concerning *Campylobacter*. The chi-square test showed significant differences in the levels of knowledge between the different age groups (*p* = 0.002), education levels (*p* = 0.013), and occupational groups (i.e., veterinarians and non-veterinarians (*p* <  0.001)), as well as between participants who were active in the food chain and those who did not work in the food chain (*p* = 0.001) (Table 5). All other variables tested, such as gender, location of main residence, state affiliation, frequency of cold cut and meat consumption, number of children in the household and household income after taxes, were not statistically significantly associated with the level of knowledge about *Campylobacter* at the univariable level (Table 5). Selected influencing factors were included in the multivariable logistic regression model. In addition to the variables that were statistically significant in the univariable analysis, no further relevant potential risk factors or confounders were identified for inclusion in the multivariable model. In the final logistic regression model, the results of the univariable tests could be confirmed. Thus, the knowledge about *Campylobacter* differed significantly between different age groups (*p* <  0.001), educational levels (*p* = 0.010) and selected professional groups, such as veterinarians and non-veterinarians (*p* = 0.004) and actors and non-actors in the food chain (*p* = 0.007). Knowledge increased significantly with age: 20- to 39-year-old participants were approximately twice as likely to know about *Campylobacter* as 16- to 19-year-old participants (OR = 2.021). The 40- to 59-year-old participants were slightly more than 2.5 times more likely to know about *Campylobacter* than the comparison group of 16- to 19-year-olds (OR = 2.664). The participants older than 60 years old were almost 3 times more likely to know about *Campylobacter* than the comparison group (OR = 2.982). We could also show that consumers with a higher level of education were significantly more frequently informed than those with a lower level of education. Consumers who had at least a secondary school certificate were almost twice as likely to know about *Campylobacter* as those who had no school certificate or a lower secondary school certificate (OR = 1.899). Participants who were not actors in the food chain were significantly less frequently informed about *Campylobacter* than those in the food chain. This also applies to non-veterinarians in comparison to veterinarians.

**Table 5:** General level of knowledge about Campylobacter among consumer groups based on a representative survey in Germany (2017)

	n total	n (%) *Campylobacter* is known	n (%) *Campylobacter* is unknown	X^2^ test	*p*-value	Multivariable logistic regression (*n* = 986)			
						b(SE)	*p*-value	Adj. OR	95% CI
**Gender**				0.509	0.476				
Female	500	164 (32.8)	336 (67.2)						
Male	508	156 (30.7)	352 (69.3)						
**Age group**				15.356	**0.002**				
16–19 years old	176	35 (19.9)	141 (80.1)				< 0.001	baseline	
20–39 years old	219	68 (31.1)	151 (68.9)			0.704 (0.256)	0.006	2.021	1.223–3.341
40–59 years old	451	158 (35.0)	293 (65.0)			0.98 (0.231)	< 0.001	2.664	1.693–4.191
> 60 years old	162	59 (36.4)	103 (63.6)			1.093 (0.267)	< 0.001	2.982	1.768–5.030
**Federal state**				0.383	0.536				
Eastern Germany	215	72 (33.5)	143 (66.5)						
Western Germany	793	248 (31.3)	545 (68.7)						
**Cold cut consumption/week**				4.030	0.133				
No consumption (never)	47	10 (21.3)	37 (78.7)						
Rare to frequent (< 1/week up to 3–4/week)	582	179 30.8)	403 69.2)						
Very common (5–6/week or daily)	379	131 (37.6)	248 (65.4)						
**Meat consumption/week**				4.377	0.112				
No consumption (never)	35	10 (28.6)	25 (71.4)						
Rare to frequent (< 1/week up to 3–4/week)	784	238 (30.4)	546 (69.6)						
Very common (5-6x/week or daily)	189	72 (38.1)	117 (61.9)						
**Level of education**				6.219	**0.013**				
No certificate or lower secondary school certificate	108	23 (21.3)	85 (78.7)					baseline	
At least secondary school certificate	878	291 (33.1)	587 (66.9)			0.641 (0.521)	0.01	1.899	1.162–3.104
**Size of the main residence (number of inhabitants)**				2.328	0.507				
Rural community (< 5000)	158	47 (29.7)	111 (70.3)						
Small-size town (between 5000 and less than 20,000)	220	63 (28.6)	157 (71.4)						
Mid-size town (between 20,000 and less than 100,000)	264	89 (33.7)	175 (66.3)						
Metropolis (100,000 and more)	332	112 (33.7)	220 (66.3)						
**Children in the household**				< 0.001	0.984				
At least 1 children	337	108 (32.0)	229 (68.0)						
No children	654	210 (32.1)	444 (67.9)						
**Monthly household income after taxes**				3.185	0.203				
Low income (< 1500€)	252	73 (29.0)	179 (71.0)						
Middle income (1500 - < 3600€)	418	141 (33.7)	277 (66.3)						
High income (> 3600€)	176	65 (36.9)	111 (63.1)						
**Occupational groups**				11.221	**0.001**				
Actor in the food chain	117	53 (45.3)	64 (54.7)					baseline	
Not an actor in the food chain	891	267 (30.0)	624 (70.0)			−0.589 (0.217)	0.007	0.555	0.362–0.849
**Veterinarian**				14.916	**< 0.001**				
Yes	12	10 (83.3)	2 (16.7)					baseline	
No	996	310 (31.1)	686 (68.9)			−2.381 (0.817)	0.004	0.092	0.019–0.459

### Knowledge differences about meat as a vector of *Campylobacter* within different consumer groups

In the univariable data analyses that focused on consumer knowledge of the transferability of *Campylobacter* via meat as the target variable, we could show a statistically significant effect of age (*p* = 0.042). All other variables examined did not show significant associations (Table 6). Only participants who knew about *Campylobacter* (*n* = 320) were included in this analysis. The logistic regression confirmed that only age group was statistically associated with consumer knowledge of *Campylobacter* transmissibility via meat (*p* = 0.044), although pairwise comparisons among age groups did not reveal significant differences (Table 7).

**Table 6:** Knowledge of consumer groups about the transmissibility of Campylobacter via meat based on a representative survey in Germany (2017). Included were participants who knew about Campylobacter (*n* = 320)

	n total	n (%) Transferability is known	n (%) Transferability is unknown	X^2^ -test *p*-value
**Gender**				0.926
Female	164	86 (52.4)	78 (47.6)	
Male	156	81 (51.9)	75 (48.1)	
**Age group**				**0.042**
16–19 years old	35	16 (45.7)	19 (54.3)	
20–39 years old	68	34 (50.0)	34 (50.0)	
40–59 years old	158	94 (59.5)	64 (40.5)	
> 60 years old	59	23 (39.0)	36 (61.0)	
**Federal state**				0.516
Eastern Germany	72	40 (55.6)	32 (44.4)	
Western Germany	248	127 (51.2)	121 (48.8)	
**Cold cut consumption/week**				0.083
No consumption (never)	10	8 (80.0)	2(20.0)	
Rare to frequent (< 1/week up to 3–4/week)	179	86 (48.0)	93 (52.0)	
Very common (5–6/week or daily)	131	73 (55.7)	58 (44.3)	
**Meat consumption/week**				0.506
No consumption (never)	10	7 (70.0)	3 (30.0)	
Rare to frequent (< 1/week up to 3–4/week)	238	122 (51.3)	116 (48.7)	
Very common (5-6x/week or daily)	72	38 (52.8)	34 (47.2)	
**Level of education**				0.615
No certificate or lower secondary school certificate	23	11 (47.8)	12 (52.2)	
At least secondary school certificate	291	155 (53.3)	136 (46.7)	
**Size of the main residence**				0.511
Rural community (< 5000 inhabitants)	47	26 (55.3)	21 (44.7)	
Small-size town (5000 - < 20,000 inhabitants)	63	34 (54.0)	29 (46.0)	
Mid-size town (20,000 - < 100,000 inhabitants)	89	41 (46.1)	48 (53.9)	
Metropolis (100,000 inhabitants and more)	112	63 (56.3)	49 (43.8)	
**Children in the household**				0.519
At least 1 child	108	54 (50.0)	54 (50.0)	
No children	210	113 (53.8)	97 (46.2)	
**Monthly household income after taxes**				0.602
Low income (< 1500€)	73	43 (58.9)	30 (41.1)	
Middle income (1500€ to < 3600€)	141	75 (53.2)	66 (46.8)	
High income (> 3600€)	65	33 (50.8)	32 (49.2)	
**Occupational groups**				0.918
Actor in the food chain	53	28 (52.8)	25 (47.2)	
Not an actor in the food chain	267	139 (52.1)	128 (47.9)	
**Veterinarian**				0.888
Yes	10	5 (50.0)	5 (50.0)	
No	310	162 (52.3)	148 (47.7)

**Table 7:** Differences by age group in the knowledge that *Campylobacter* is transmissible via meat

Logistic regression (*n* = 320)				
	b(SE)	*p*-value	OR	95% CI
16–19 years old		**0.044**		
20–39 years old	0.172 (0,417)	0.680	1.188	0.524–2.689
40–59 years old	0.556 (0,376)	0.139	1.744	0.835–3.645
> 60 years old	−0.276 (0,432)	0.522	0.759	0.326–1.768

### Knowledge differences about meat as a vector of *Campylobacter*, *Salmonella* and *Toxoplasma* between consumers with different levels of knowledge about possible protective measures against these pathogens (Tables 2, 3 and 4)

The chi-square test proved a significant difference in knowledge about the transferability of *Campylobacter* via meat between consumers with different levels of knowledge about possible protective measures against *Campylobacter* (*p* = 0.002). Those who knew how to protect themselves from *Campylobacter* infection were approximately twice as likely to know that *Campylobacter* was transmitted via meat as those who knew about *Campylobacter* but did not know how to protect themselves (OR = 2.103).

With regard to *Salmonella,* the results indicated that there was a significant difference in knowledge about the transmissibility of *Salmonella* via meat between consumers with different consumer knowledge about possible protective measures against *Salmonella* (*p* <  0.001). Those who knew how to protect themselves against *Salmonella* infection knew about twice as often that *Salmonella* was transmitted via meat as those who knew about *Salmonella* but did not know how to protect themselves (OR = 2.1).

With regard to *Toxoplasma,* it was shown that there was a significant difference in knowledge about the transferability of *Toxoplasma* via meat between consumers with different levels of knowledge about possible protective measures against *Toxoplasma* (*p* <  0.001). Those who knew how to protect themselves against *Toxoplasma* infection knew approximately 2.5 times more often that *Toxoplasma* was transmitted via meat than those who knew about *Toxoplasma* but did not know how to protect themselves (OR = 2.6).

## Discussion

Campylobacteriosis was the most frequently reported zoonosis throughout the European Union in 2017, and salmonellosis was the second-most common zoonosis to be reported in the European Union [2]. In addition to Europe, the number of cases of campylobacteriosis has also increased in North America and Australia [21]. The significant increase in the number of cases from below 55,000 in 2001 to more than 70,000 in 2016 in Germany highlights the importance of raising consumer awareness of *Campylobacter*. One reason for the increased case numbers could be that human consumption of poultry meat in Germany increased from 10.92 kg/head in 2001 to 13.19 kg/head in 2018 [22], and consumers therefore come into contact with *Campylobacter* more frequently. Another reason for the increasing case numbers, especially in the summer months from June to September, could be the increased ambient temperature. Yun and colleagues [23] showed that the increase in ambient temperature is positively associated with the occurrence of *Campylobacter*. Although the incidences of salmonellosis decreased from 2001 to 2016 in Germany, in 2018, the number of infections was higher than in 2016 [24]. This shows that the success of activities to reduce the incidence cannot be taken for granted. The incidence of clinical toxoplasmosis ranged from 6 to 23 cases in the years 2002–2018 [24] and thus remains well below the incidence of *Salmonella* and *Campylobacter*.

Since this survey was conducted by a commercial online survey company and included target panels with a stratified sample, the survey could be conducted with little effort in a short amount of time. The number of necessary participants was quickly reached, and time-consuming recruitment was not necessary. In addition, the acceptance among the participants was high, as they consciously decided to be participants in a panel, and the questionnaire could be answered online at any time of day and at any place. Data input and transmission were performed automatically so that transmission errors could be minimized. However, this did not insulate the study from forms of bias that are characteristic of online surveys. First, not all participant groups may be available online. In the event of queries, assistance may not be possible. In addition, the environment cannot be controlled during the survey. The presence of third parties cannot be ruled out, nor can the presence of other media, e.g., to provide assistance, be excluded [25]. Answering individual questionnaires by using automated answer scripts is theoretically possible but rather unlikely. The survey is not open to the public, and each person receives an individual e-mail link that can only be used once. Although there is no 100% guarantee, the panel provider takes as much care as possible to prevent automated answers.

Our results showed that the proportion of participants who did not know about *Campylobacter* at all was 68.3%. This corresponds with the results of another study among consumers from Germany, in which 75% of the respondents did not know that *Campylobacter* occurs in food [9]. Publications from other European and non-European countries also show that consumers’ knowledge of *Campylobacter* is predominantly poor. A total of 83.3% of Slovenian consumers did not know about *Campylobacter* [19]. In an Australian study, only 8% of respondents knew about *Campylobacter* [16]. A total of 22% of respondents in an Austrian study [14] and 16% of respondents in a U.S. study [13] had already heard of *Campylobacter*. In the U.S., consumer knowledge seems to have increased in recent years, since in an earlier study, only 7% of the participants had heard of *Campylobacter* [15].

Our results also showed that most consumers in Germany (> 97%) have heard of *Salmonella* at least once before. This corresponds with the results of another study among consumers from Germany, in which 96% of consumers had already heard of *Salmonella* in food [26]. In another study with an open question about pathogenic germs in food, only slightly more than half (58.3%; *n* = 420) of the respondents mentioned *Salmonella* [27]. Publications from other European and non-European countries also showed that consumer knowledge of *Salmonella* is generally good. In Austria, 98% of consumers knew about *Salmonella* [14]. In Ireland, 92.9% of respondents have already heard of *Salmonella* [18]. In the Netherlands, a study found that 97.4% of respondents said they knew that they could be infected with *Salmonella* from contaminated foods [28]. In two U.S. studies, more than 90% of consumers had already heard of *Salmonella* [13, 15]. In comparison to *Salmonella* and *Campylobacter*, our results showed that the general knowledge about *Toxoplasma* was almost equally divided among consumers. Forty-eight percent had never heard of the pathogen, and 51.7% had at least heard of *Toxoplasma*. In a study from Poland in which 565 pregnant women participated, 439 (94.4%) of the respondents were aware of toxoplasmosis. A total of 77.4% knew it was a zoonosis [29]. A U.S. study showed that 48% of pregnant women had heard or seen information about toxoplasmosis [30]. A survey of pregnant and postpartum women in Brazil showed that only 27.8% knew that the disease existed. Most of them (72.2%) had never heard of toxoplasmosis [31]. In a study from Zimbabwe, only 4% of 49 respondents knew that toxoplasmosis was a zoonosis that could be transmitted via cats [32]. Overall, there appear to be fewer consumer surveys than on *Campylobacter* and *Salmonella*. This may be because clinical symptoms usually do not occur except in pregnant women. In addition, the overall case numbers are significantly lower than those for *Campylobacter* and *Salmonella* in Germany as well as throughout the EU [33].

Our study showed that meat was not sufficiently known as the main vector of *Campylobacter*. Only half (52.2%) of those who knew about *Campylobacter* (*n* = 320) knew that it could be transmitted to humans via meat. Although 116 consumers indicated that they knew how they could protect themselves, 36.2% (42/116) did not know that transmission occurs via meat. In general, consumers are aware that food-borne infections are often associated with chicken meat [34, 35], but an international comparison also shows that consumers do not know that meat is a vector of *Campylobacter*. An Australian study showed that only 9% of consumers associate *Campylobacter* with chicken and poultry [16]. In a U.S. study, only 0.4% of respondents could name a *Campylobacter* vector [36]. In Slovenia, only 18% of respondents knew how often *Campylobacter* was present on poultry meat in retail outlets [19]. A study from Switzerland showed a high level of general knowledge about pathogenic bacteria in poultry meat, but pathogenic bacteria are perceived as the least threatening in comparison to other potential food risks, such as the intake of too many calories, an unbalanced diet, hormone residues in meat or allergies [37]. In New Zealand, only 15% of respondents knew that a very high proportion of fresh chicken is contaminated with *Campylobacter* [11]. In a UK study, 24% of respondents had heard that *Campylobacter* can cause foodborne infection [12].

Our study also showed that meat was predominantly known as a vector of *Salmonella*. Only 13.4% of all respondents (131/980) who knew about *Salmonella* said they did not know that these pathogens could be transmitted to humans via meat. Nevertheless, it was found that some consumers misjudged their knowledge. Of those who said they knew how to protect themselves, 11.3% (88/779) did not know that *Salmonella* was transmissible via meat. An international comparison shows that many consumers are aware that meat can be a source of *Salmonella* transmission. A survey of students at the University of Maine showed that slightly more than half of those surveyed (57.3%) were aware of an association between *Salmonella* and raw chicken [38]. Murray and Glass-Kaastra [34] showed that the majority of respondents are aware of the risks of foodborne illness associated with chickens, and the majority are aware that chickens that are not fully cooked can be a cause of foodborne illness. A study in Mexico showed that fresh meat is the most commonly considered sources of salmonellosis compared to other food categories, such as “fruits and vegetables” or “dairy products” [39]. In an Italian study, on the other hand, the awareness of *Salmonella* transmission was not particularly high. Only approximately ¼ of the respondents were aware of food vehicles for the transmission of *Salmonella* [40]. In Ireland, a study showed that of those who knew about *Salmonella*, only 23.1% knew that *Salmonella* can be transmitted via poultry. Only 4.7% knew that *Salmonella* could be transmitted via pork. The most frequently mentioned possible vectors that were correct were eggs (44%) [18].

In our study, 50.7% knew that *Toxoplasma* could be transmitted via meat. Thus, we can conclude that consumers know more about the transmissibility of *Toxoplasma* via meat than about the transmissibility of *Campylobacter* via meat. A U.S. study showed that only 30% of pregnant women were aware that Toxoplasma may be found in raw or undercooked meat [30]. Another U.S. showed that only 24% of the respondents knew that *Toxoplasma* can be transmitted via food [13]. In a study from Poland that included only pregnant women, 46.7% knew that raw or uncooked meat was a route of transmission [29]. Nevertheless, it is also evident that significantly fewer consumers (16%) know that *Campylobacter* can be transmitted via food. Again, most consumers (93%) know that *Salmonella* can be transmitted via food [13].

Since the consumption of meat is known to be the main cause of C*ampylobacter* infection, a reduction in meat consumption could lead to a reduced incidence of *Campylobacter* food-borne infections. A general reduction in meat consumption would also have the advantage of a lower number of *Salmonella* and *Toxoplasma* infections, although consumer knowledge of these pathogens is higher. Clinically manifest diseases or even deaths associated with the consumption of meat, and therefore secondary health care costs, may be reduced if knowledge about foodborne diseases were more widespread. Switching to a vegetarian diet would also reduce infection with these pathogens, as meat is the most common source of foodborne infections. A complete reduction in incidence is not possible because *Campylobacter* is also transmissible through raw milk [3] and *Salmonella* through eggs [33].

In addition, only 11.5% of the participants in our study who had heard of at least *Campylobacter* (*n* = 320) knew how to protect themselves from *Campylobacter* infection. Thus, it is not sufficient only to increase the level of knowledge about *Campylobacter*. In Germany, there are still too many consumers who do not wash their hands or the cutting board after preparing raw meat [41]. This result seems contradictory at first, since a survey of the German Federal Institute for Risk Assessment shows that 90% of the respondents indicated that they know how to protect themselves against pathogenic bacteria in their own household. At the same time, this survey also shows that only a minority of 9% of German consumers believe that compliance with kitchen hygiene serves as a protective measure against bacteria [42]. International comparative studies show that consumers are well aware of good hygiene practices and that many consumers are familiar with hygiene measures, such as washing their hands after handling raw meat [43, 44]. One reason for the nevertheless increasing incidence of foodborne infections in general could be that consumers do not wash their hands properly, and cross-contamination still occurs [45]. Health policy has long recognized that insufficient consumer awareness of *Campylobacter* is a problem, and scientific institutions have already compiled comprehensive information for consumers. However, although much information about *Campylobacter* and protection against infection is available at the national and international levels [46–49], our results suggest that the available information does not reach consumers. Consumers must actively search for available information material. Increased media attention could increase consumer awareness and vigilance in food handling [44]. The general lack of dramatic outbreak situations for *Campylobacter* explains why media attention is rather weak. The total number of *Campylobacter* outbreaks is much lower than that of S*almonella* infections. The number of people who need hospital treatment due to clinical symptoms is much lower for *Campylobacter* than for S*almonella* [33]. Regarding *Campylobacter*, 3% of the patients need to be hospitalized, whereas this is necessary for 19.5% of *Salmonella* patients. In addition, low mortality has occurred in those with *Campylobacter* infections than in those with *Salmonella* infections [33].

Providing a label with appropriate handling instructions or warning signs indicating the *Campylobacter* risk could increase consumer awareness. This is confirmed, for example, by the results of other consumer surveys [11, 50]. Approximately 80% of consumers in Germany have the opinion that it is not easy to discern whether a foodstuff can cause health problems if handled incorrectly [51]. According to the results of the Deutsche Landwirtschafts-Gesellschaft e.V [52]., which informs consumers about the quality of food (among other things) and conducts studies on the food industry, consumers in Germany think that such information on the packaging would be very useful. It is therefore necessary to identify methods to ensure that the available information materials reach consumers.

According to the Robert Koch Institute case numbers of *Campylobacter*, men are generally more exposed to infections than women [53]. However, in our study, we could not show that men were significantly less informed about *Campylobacter* than women. Therefore, we could not confirm any association between the level of knowledge of men and women and the incidence of the disease. This is also coincident with results from Lin et al. [15]. Women are significantly more interested in food safety issues than men, although there is no statistically significant relationship between gender and food safety knowledge [35]. This is confirmed in other studies [54, 55]. Rossvoll et al. [56] came to a different conclusion: according to their results, men seem to know less about food safety than women and have more knowledge deficits in hygiene practices. It has also been shown that there are knowledge differences between men and women regarding the fact that microorganisms are the cause of food-borne infections [57]. Tomaszewska et al. [58] found different results in two different countries in their study: while in Poland, women showed a slightly higher level of knowledge about food hygiene than men, this significant gender difference in knowledge could not be established in Thailand.

Younger consumers are less interested in food safety issues than older consumers are [35]. This could explain why the knowledge about *Campylobacter* in our study differed statistically significant by age group, and young adults < 20 years were the least informed. A possible explanation would be that approximately 80% of the female and over 80% of the male 19-year-olds still live in their parents’ households [59]. In addition, 30% of individuals under 19 years old in Germany generally do not prepare their meals themselves, whereas those over 60 years old cook more often than the average person [60]. Children may not be as concerned about food safety and the transmission of pathogens through food because the parents often prepare the food for the children even if they are already grown up. In comparison to our study, Lin et al. [15] found that the age groups investigated in their study did not differ significantly with regard to *Campylobacter* knowledge. Similarly, Stratev et al. [54] could not establish a significant relationship between age and knowledge of food safety.

We found that consumers with a higher education level were significantly more informed about *Campylobacter* than those with lower education. Similar results were also shown in a U.S. study. Consumers with at least some college education are more likely to have heard of *Campylobacter* than those with less education were [15]. Further study results also suggest that the more educated consumers are, the better their knowledge of food safety [55, 61]. However, Zorba and Kaptan [35] found no significant correlation between educational level and food safety issues.

Comparable to our study, other studies have not found that consumers with higher household incomes after taxes are significantly better informed about *Campylobacter* than are consumers with lower incomes [15]. However, there are also studies showing that safe food handling is more prevalent among consumers with higher incomes [62].

We could confirm that actors in the food chain, and veterinarians in particular, are better informed about *Campylobacter* than are those who are not or have not been active in this sector. We have assumed that there is a certain level of knowledge about pathogens that occur in the immediate occupational sector. A study from Ontario that surveyed actors and veterinarians involved in pig production showed corresponding results. More veterinarians were familiar with *Campylobacter* and other microbial hazards than were individuals in other occupational groups. One explanation seems to be that veterinarians are informed about pathogens through their education and that knowledge about zoonosis is an important component [63]. However, our finding that more than half of the actors in the food chain (54.7%) did not know about *Campylobacter* at all was very interesting. One survey showed significant gaps in the knowledge of *Campylobacter* among broiler chicken producers. While 82.4% of those surveyed know that *Salmonella* could be transmitted to humans via contaminated chicken meat, only 21% of chicken meat producers knew that the same applies to *Campylobacter* [64]. A survey of pork producers showed that knowledge of *Campylobacter* is also low among this group. Only 12.8% of respondents knew that *Campylobacter* could infect humans [65]. There also seem to be gaps in the knowledge of food workers in meat processing plants. While there is a high level of knowledge of general protective measures, most workers are not well aware of specific diseases or pathogens that could be transmitted through food [66]. A U.S. study from Pennsylvania also showed gaps in the knowledge of poultry product vendors about pathogens and cross-contamination during poultry processing [67].

Although there are statistically significant differences in the level of knowledge by age group, educational level, occupational group and status as a veterinarian, there must be other factors that significantly influence the level of knowledge.

## Conclusions

*Campylobacter*, despite its high incidence in Germany, is largely unknown to consumers. Since elimination from poultry farms and within the poultry production chain is not foreseeable at present, one focus of infection prevention and educational work must be to sensitize consumers. Based on the results of our study, it can be concluded that the risk of a foodborne infection by *Campylobacter* may be underestimated or not perceived as such, and consumers’ assessments do not seem to correspond to scientific findings. Even if certain consumer groups appear to be better informed than others, it is evident, nationally and internationally, that consumer knowledge about *Campylobacter* and their transmission routes must be increased to reduce the high annual incidence of *Campylobacter* infections. However, the findings seem to be different for *Salmonella*. Knowledge about *Salmonella* is much better, and the number of cases has decreased since the beginning of this century. Although knowledge about toxoplasmosis is not as high as knowledge about *Salmonella*, the persons for whom an infection is clinically relevant seem to be well informed. Overall, it is a great challenge to accurately target information on the safe handling of food to consumers. There is no lack of information materials per se, and educational campaigns take place in the real world as well as in social media. Consumers must become aware that they have a large part of the responsibility themselves. It must be made clear that purchased food might contain pathogenic microorganisms and that it is up to consumers to safely handle food or prepare food to kill microorganisms before consumption. In addition to heating raw meat sufficiently, consumers must comply with general hygiene measures, such as washing hands, and reduce cross-contamination by using various kitchen utensils. Finally, the risk of infection can be reduced by reducing meat consumption. Educating consumers about the responsibilities of the actors in the food chain, including themselves, could help to reduce foodborne infections. To provide information materials on the abovementioned risks and protective measures against food-borne infections to consumers, one possibility would be to disseminate information via social media. This would enable a large number of consumers to be reached, as such information would be passed on to friends and acquaintances by the consumers themselves. The development of an innovative phone app would also be conceivable, since a large part of the population is now reached through this medium; gaps in knowledge could be conveyed through this phone app. Appropriate marketing at the point of sale, in newspapers, in social media and on TV would be useful here, so that as many consumers as possible are made aware of such an app.

## Data Availability

Raw data of the study are available upon request to the corresponding author.

## Abbreviations

OR: Odds ratio
US: United States
e.g.: Example given
